# High Serum Tumor Necrosis Factor-Alpha Levels in Women with Polycystic Ovary Syndrome: A Meta-Analysis

**DOI:** 10.1371/journal.pone.0164021

**Published:** 2016-10-20

**Authors:** Lingling Gao, Yang Gu, Xianghua Yin

**Affiliations:** Department of Obstetrics and Gynecology, Clinical Medical College of Yangzhou University (Subei People's Hospital of Jiangsu Province), Yangzhou, Jiangsu, China; Weill Cornell Medical College Qatar, QATAR

## Abstract

The objective of the study is to assess the TNF-α levels in PCOS patients and healthy controls. A comprehensive electronic search in Medline, Embase, and the Cochrane Library database was conducted up to July 2016. Random-effects model was used to estimate the standardized mean differences (SMDs) with 95% confidence intervals (CIs). Twenty-nine studies with a total of 1960 participants (1046 PCOS patients and 914 controls) were included in this meta-analysis. The TNF-α levels in PCOS patients were significantly higher than those in controls (random-effects, SMD = 0.60, 95% CI = 0.28–0.92, P<0.001). With regard to the subgroup analyses stratified by ethnicity, study quality, methods, and BMI, significantly high TNF-α levels were found in patients with PCOS in almost all of these subgroups. In the subgroup stratified by HOMA-IR ratio and T ratio, significant differences were only observed in the subgroups with HOMA-IR ratio of >1.72(SMD = 0.967, 95% CI = 0.103–1.831, P = 0.028, I^2^ = 93.5%) and T ratio>2.10 (SMD = 1.420, 95% CI = 0.429–2.411, P = 0.005, I^2^ = 96.1%). By meta-regression it was suggested that ethnicity might contribute little to the heterogeneity between the included studies. Through cumulative meta-analysis and sensitivity analysis it was supposed that the higher TNF-α levels of PCOS patients compared to healthy controls was stable and reliable. This meta-analysis suggests that the circulating TNF-α levels in women with PCOS are significantly higher than those in healthy controls. It may be involved in promoting insulin resistance and androgen excess of PCOS.

## Introduction

Polycystic ovary syndrome (PCOS) is one of the most common heterogeneous endocrine disorders, which affects 5%–10% of women in reproductive age and is considered as one of the leading causes of female infertility[1]. It is characterized by biochemical or clinical hyperandrogenism, polycystic ovaries on ultrasonography and oligo-ovulation or anovulation. Besides, PCOS frequently accompanies with metabolic abnormality such as insulin resistance and obesity which predisposes women with PCOS to type 2 diabetes mellitus(T2DM)[2] and cardiovascular disease[3].

PCOS is also a proinflammatory state. Low-grade chronic inflammation in women with PCOS is involved in the pathogenesis of T2DM and cardiovascular disease[4]. Tumor necrosis factor-alpha (TNF-α) is a major proinflammatory cytokine and expressed mainly in monocytes, macrophages and adipose tissue. Serum levels of TNF-α were elevated in both obesity and T2DM [5]. TNF-α played a role in the pathogenesis of insulin resistance [5]. It inhibited tyrosine phosphorylation of insulin receptor and insulin receptor substrate-1 in muscle and fat cells [5]. Besides, it down-regulated the expression of the glucose transporter type 4 which was necessary for cellular transport of glucose [6]. TNF-α might also play a key role in the development of cardiovascular disease. Elevated levels of TNF-α were reported to be associated with an increased risk of future myocardial infarction[7]. Therefore, TNF-α may be a key mediator which is linked to T2DM and cardiovascular diseases in women with PCOS. Therefore, TNF-α may be a useful biomarker for the diagnosis of PCOS and the treatment of T2DM and cardiovascular diseases in women with PCOS.

Until recently, a number of studies have investigated the changes of TNF-α levels in PCOS patients. However, the results of these studies were contradictory rather than conclusive. Some studies reported significant elevation of TNF-α levels in PCOS women compared with healthy controls[8–15], but these were not confirmed in similar studies[16–28], with some studies even reporting decreased TNF-α levels[29]. Two meta-analysis of comparing circulating TNF-α levels in women with PCOS and healthy controls were reported in 2011[30, 31]. The analysis by toulis et al[31] revealed that TNF-α levels were higher in women with PCOS than in controls, but the other meta-analysis found no significant difference in TNF-α levels of PCOS women and controls[30]. During the last 5 years, many more relevant studies have been published and presented inconsistent results [11–15, 23–28]. Moreover, those two meta-analysis did not report the relations between TNF-α levels and the characteristics of PCOS, such as body mass index (BMI), insulin resistance, and androgen status. Thus, a updated meta-analysis of the literature was conducted to investigate the circulating TNF-α levels in PCOS women compared to healthy controls and the relations between TNF-α levels and the characteristics of PCOS.

## Material and Methods

### Search Strategy

A comprehensive electronic search in Medline, Embase, and the Cochrane Library database was conducted from inception to July 2016, using both free words and index terms specific to each search platform (Emtree in Embase.com and MeSH in Cochrane Library). The search strategies were based on combinations of the keywords: ‘tumor necrosis factor-alpha’, ‘TNF-alpha’, ‘tumor necrosis factor-α’, ‘TNF-α’ coupled with ‘polycystic ovary syndrome’, or ‘PCOS’. The detailed search strategies are listed in supplementary data (S1 File). The electronic investigation was supplemented with a manual search of references of all articles retrieved. The latest searches were undertaken to 1 July 2016. There were no language restrictions. Two reviewers independently searched the electronic databases, and screened the titles, abstracts, and full-text after excluding duplicated studies. Any discrepancy in the screening process was resolved by consultation of the group.

### Study selection

Studies were included in the analysis if: 1. the study reported circulating TNF-α levels in women with PCOS and healthy women controls. 2. the study provided TNF-α means (M) and standard deviation (SD) or sufficient information to calculate them. Letters, case reports, editorials, and conference abstracts were excluded. Additionally, studies in which patients with diseases more than PCOS, as well as studies involving pregnant patients were also excluded.

### Quality Score Assessment

Quality evaluation of included studies was conducted according to the Newcastle-Ottawa Scale[32] with minor modification. The quality assessment criteria used in the study were: 1. whether the diagnosis of the PCOS was with independent validation; 2. whether the involved cases were representative of population; 3. whether the controls enrolled were from the same community; 4. whether the controls were described to have no history of disease; 5. whether the cases and controls were matched for age or BMI; 6. Whether the cases and controls were matched for additional factor, such as drinking or smoking status; 7. whether the sample size was >50. For each criterion a score of 0 or 1 was assigned according to whether the criterion was satisfactorily fulfilled. According to the quality score assessment, the distribution of the scores was between 0 and 6. Studies with score of 5 or above are classified as high quality studies. Others are categorized as low quality studies.

### Data Extraction

Two of the authors independently extracted the data from the included studies. The general characteristics of the study were extracted using a standardized data extraction form. The general characteristics (name of the first author, year of publication, country, diagnostic criteria, matched factors, number of the PCOS group and control group) were listed. Further, the following data of the PCOS and control groups were extracted and double-checked: methods used to measure TNF-α levels, BMI, age, TNF-α levels, insulin sensitivity status, and total testosterone levels. If the two investigators could not reach an agreement, the dispute was resolved by a third reviewer.

### Statistical Analysis

TNF-α levels were extracted as M±SDs in each study. Missing SDs were calculated by reported standard error. Where appropriate, the data was completed through communication with the authors. Homeostasis Model Assessment of Insulin Resistance (HOMA-IR) values were used to measure the degree of insulin resistance. When not reported, missing HOMA-IR values were calculated on the basis of mean fasting glucose and insulin values, using the Oxford Diabetes Trials Unit calculator (www.dtu.ox.ac.uk). The relative between-group difference in insulin resistance was expressed as the ratio of mean HOMA-IR value in the PCOS group to that of the controls. Similarly, the relative difference in androgen status was expressed by the ratio of mean total testosterone (T ratio) in PCOS women to controls. Standardized mean differences (SMDs) with 95% confidence intervals (CIs) was used to estimate the effect size.

Heterogeneity across studies was quantified using the Q-statistic and inconsistency index (I^2^). If *P* < 0.10, the heterogeneity was considered as statistically significant. If I^2^> 50%, it was considered as large heterogeneity; if I^2^ = 25–50%, as moderate heterogeneity and if I^2^ < 25%, as absent of heterogeneity. In case of large heterogeneity (I^2^>50%), random-effects model was applied since it was usually more conservative. Otherwise, a fixed-effects model was used. When the results revealed statistically significant heterogeneity, possible explanations were investigated by subgroup analysis according to the following factors: ethnicity, quality score, methods used to measure TNF-α levels, BMI, HOMA-IR ratio, and T ratio. The BMI was categorized into two groups: a lean group (BMI <25 kg/m^2^) and an obese group (BMI ≥25 kg/m^2^). The HOMA-IR ratio and T ratio were categorized according to their quartile intervals. A meta-regression analysis was performed to investigate the sources of heterogeneity. The SMD was used as the dependent variable, and the year, ethnicity, sample Size, quality score, BMI, age, HOMA-IR ratio, T ratio, and sample size were used as explanatory covariates. A multivariable analysis was further performed if more than one variable were significant at the 0.1 level. A sensitivity analysis, with the studies omitted one by one, was performed to examine the influence of individual studies. A cumulative sequential meta-analysis of the studies was performed according to their year of publication. To assess the publication bias bias, begg’s test, egger’s test and trim-and-fill was applied. All statistical analyses were carried out with STATA software (version 10.0; Stata Corporation, College Station, TX).

## Results

### Literature Selection

**Fig 1:**
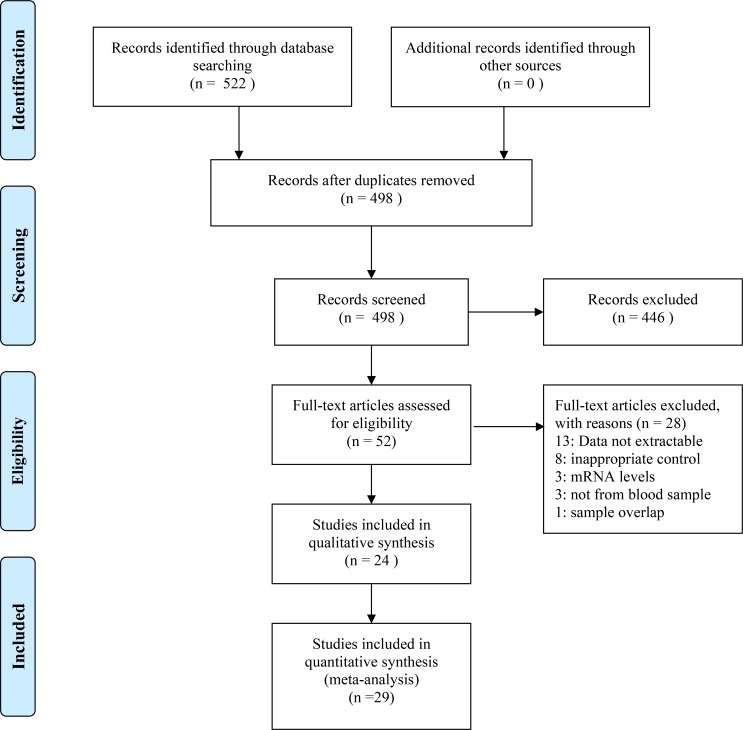
Flow chart showing the study selection for this meta-analysis.

A flow chart showing the study selection is presented in Fig 1. Using the search strategy predefined 522 potentially relevant studies were identified. After removing duplicated records and reviewing the titles and abstracts, 52 potential eligible studies that evaluate the TNF-α levels in PCOS and controls were identified for full-text assessment. Of these, 28 articles that did not fulfill the selection criteria were excluded(S2 File). Ten studies were excluded because the data was asymmetric distributed and presented by median and range[33–42]. Three studies were excluded because the data was presented by bar graph and unable to be obtained from the authors[43–45]. The control group of eight excluded studies was inappropriate[46–53]. In three excluded studies the levels of TNF-α were not tested in blood sample[54–56] and in another three studies the mRNA level of TNF-α in adipose tissue was tested[57–59].The subjects of two studies were overlapped[14, 60], so one of them was omitted[60]. Since several studies reported the data separately according to different categories of BMI (<25 kg/m^2^ or ≥25 kg/m^2^)[25, 27, 28, 61, 62], these five articles were separated as ten studies. Ultimately, 24 articles (29 studies) were included in this meta-analysis[8–29, 61, 62].

### Systematic review

The involved studies enrolled 1960 participants (1046 PCOS patients and 914 controls). The main characteristics of the included studies are demonstrated in Table 1. These studies were published between 1999 and 2015. The majority of the studies were conducted in Asia, whereas six were done in America, five in Europe, and one in Australia. Nineteen of the reports were conducted in Caucasian population, and ten study were conducted in Asian population (S2 Table). For the diagnosis of PCOS, 16 of the studies used Rotterdam criteria, 11 used National Institutes of Health (NIH) criteria, and another 2 used Androgen Excess Society (AES) criteria. The major matched factors for the PCOS and control group were BMI, age, waist circumference, and smoking. Ten of the studies found higher TNF-α levels in the PCOS group compared with the controls, and 18 studies found no statistically significant difference. Only one study reported decreased TNF-α levels in the PCOS group.

**Table 1:** Characteristics of studies included in the meta-analysis.

Study	Year	Country	Study Design	Diagnosticcriteria	Sample size		Matched factors	TNF-α Level	Methods	PCOS	Control
					PCOS	control					
Gonzalez[61]	1999	USA	Case-control	NIH	12	20	Age,BMI	↑	ELISA	3.31±0.87	0.91±0.54
Gonzalez[61]	1999	USA	Case-control	NIH	22	20	Age,BMI	NS	ELISA	4.11±0.89	4.65±1.47
Escobar-Morreale[16]	2001	Spain	Case-control	NIH	21	27	BMI	NS	CL	7.3±3.7	5.6±4.0
Araya[8]	2002	Chile	Case-control	NIH	16	11	Age	↑	CL	6.73±2.34	4.82±1.15
Escobar-Morreale[17]	2003	Spain	Case-control	NIH	35	28	BMI,Smoking	NS	CL	3.74±1.18	3.53±1.32
Sayin[9]	2003	Turkey	Case-control	NIH	21	14	Age,BMI	↑	CL	23.67±25.57	7.58±6.66
Tarkun[10]	2006	Turkey	Case-control	Rotterdam	32	25	Age,BMI,WHR	↑	CL	5.27±1.59	4.48±0.81
Vgontzas[18]	2006	USA	Case-control	NIH	42	17	BMI	NS	ELISA	4.05±1.94	3.79±0.82
Moran[19]	2007	Australia	Case-control	Rotterdam	15	17	BMI,Smoking	NS	ELISA	6.0±5.1	5.8±3.5
Olszanecka[20]	2007	Poland	Case-control	NIH	39	34	Age,BMI,Smoking	NS	ELISA	5.5±2.0	6.8±3.5
Jakubowska[29]	2008	Poland	Case-control	Rotterdam	29	29	Age,BMI,WHR	↓	RIA	10.69±13.14	14.95±13.3
Arika[21]	2009	Turkey	Case-control	Rotterdam	39	30	Age,BMI,WC	NS	CL	11.52±5.68	13.84±10.72
Samy[62]	2009	Egypt	Case-control	Rotterdam	56	35	Age,BMI	NS	ELISA	3.72±1.26	3.66±1.02
Samy[62]	2009	Egypt	Case-control	Rotterdam	52	40	Age,BMI	↑	ELISA	6.87±1.12	3.76±1.04
Soares[22]	2009	Brazil	Case-control	Rotterdam	40	50	Age,BMI,Waist	NS	CL	10.08±7.38	12.25±6.54
Ilie[23]	2011	Romania	Case-control	AES	45	32	Age,BMI	NS	ELISA	8.2±5.49	7.26±2.54
Victor[11]	2011	Spain	Case-control	Rotterdam	39	43	Age,BMI,WC	↑	MBIA	5.1±0.9	3.2±1.8
Xiong[12]	2011	China	Case-control	Rotterdam	86	50	Age	↑	CL	2.312±1.762	1.751±1.725
Choi[13]	2012	Korea	Case-control	Rotterdam	37	33	BMI,smoking	↑	ELISA	3.19±1.19	1.79±0.78
Wang[24]	2012	China	Case-control	Rotterdam	35	35	Age,BMI,WC	NS	ELISA	4.1±0.3	3.1±0.2
Lee[25]	2013	Korea	Case-control	NIH	20	20	Age,BMI	NS	RIA	1.06±0.47	1.19±0.88
Lee[25]	2013	Korea	Case-control	NIH	20	20	Age,BMI	NS	RIA	0.98±0.52	1.05±0.59
Li[14]	2013	China	Case-control	NIH	16	18	Age,BMI,WC	↑	ELISA	68.66±35.92	30.69±16.67
Pawelczak[26]	2014	USA	Case-control	Rotterdam	23	12	Age,BMI	NS	MBIA	7.4±4.08	4.8±3.16
Thathapudi[15]	2014	India	Case-control	AES	204	204	Age	↑	ELISA	13.24±10	5.5±3.8
Agacayak[27]	2015	Turkey	Case-control	Rotterdam	15	15	Age,BMI	NS	ELISA	313±248	294±292.2
Agacayak[27]	2015	Turkey	Case-control	Rotterdam	15	15	Age,BMI	NS	ELISA	212±242.1	214.5±233
souza[28]	2015	Turkey	Case-control	Rotterdam	8	10	Age,BMI	NS	CL	7.65±4.83	6.37±1.38
souza[28]	2015	Turkey	Case-control	Rotterdam	12	10	Age,BMI	NS	CL	10.47±6.92	8.11±3

NIH, National Institutes of Health; AES, Androgen Excess Society; BMI, body mass index; WC, Waist circumference; WHR, Waist-to-Hip Ratio, NS, no significant difference; ELISA, enzyme-linked immunosorbent assay; CL, chemiluminescence; RIA, radioimmunoassay; MBIA, multiplexing bead immunoassay

Furthermore, the detailed quality score for each study was listed in S2 Table. Thirteen studies scored 7 or above were categorized as high quality studies, and the other 16 studies were classified as low quality ones. The BMI in 13 of the studies was <25 kg/m^2^, and 14 of the studies ≥25 kg/m^2^ (S2 Table). Twenty-one studies reported the HOMA-IR ratio and 22 studies reported the T-ratio between PCOS women and controls to account for a difference in TNF-α levels and a percentage of the potential variability of across-study results (S2 Table).

### Meta-Analysis

#### Pooled analysis

**Fig 2:**
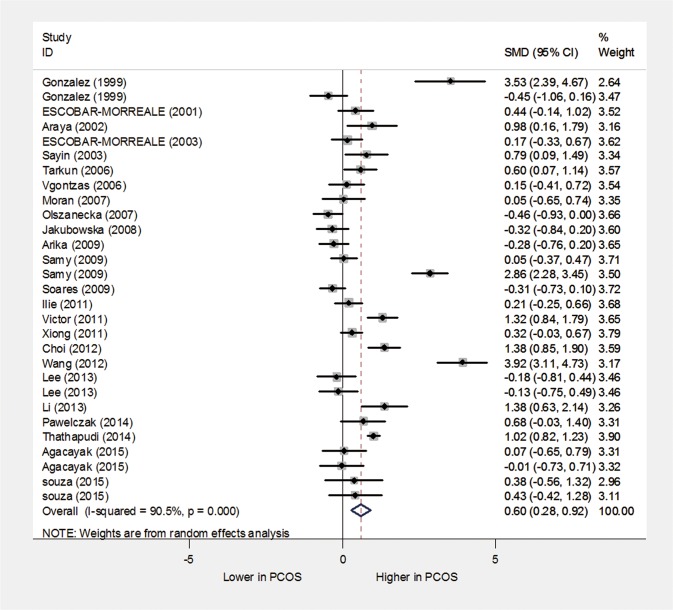
The quantitative synthesis for TNF-α levels in PCOS patients compared with controls.

The overall effect of the pooled analysis indicated that the TNF-α levels in the PCOS patients were significantly higher than in healthy controls (random-effects, SMD = 0.60, 95% CI = 0.28–0.92, P<0.001; Fig 2). However, significant heterogeneity was found across the included studies (I^2^ = 90.5% and P<0.001).

#### Subgroup analyses

Subgroup analyses were carried out according to the different categories of ethnicity, quality score, methods used to measure TNF-α levels, BMI, HOMA-IR ratio, and T ratio. The quartile intervals for the HOMA2-IR ratio were ≤1.39, 1.40–1.53, 1.54–1.89, and >1.89. The quartile intervals for the T ratio were ≤1.64, 1.65–1.81, 1.82–2.10, and >2.10. Studies with unavailable data of HOMA2-IR and total testosterone were categorized as the unknown group. The results of the subgroup analyses were shown in Table 2.

**Table 2:** Subgroup meta-analysis of TNF-α levels and polycystic ovary syndrome.

Characteristic	Studies	SMD (95% CI)	P	Heterogeneity	
				I^2^	p
Ethnicity					
Caucasian	19	0.308(0.005,0.612)	0.047	79.8%	0.000
Asia	10	1.103(0.489,1.718)	0.000	94.0%	0.000
Study quality					
Low score	16	0.360(0.070,0.650)	0.015	69.1%	0.000
High score	13	0.840(0.285,1.395)	0.003	94.8%	0.000
Methods					
ELISA	14	0.932(0.361,1.502)	0.001	94.1%	0.000
CL	10	0.284(0.020,0.548)	0.035	54.0%	0.021
RIA	3	-0.255(-0.560,0.110)	0.188	0.0%	0.883
MBIA	2	1.059(0.451,1.666)	0.001	51.4%	0.152
BMI					
<25	13	0.829(0.280,1.379)	0.003	92.2%	0.000
≥25	16	0.431(0.024,0.838)	0.038	89.3%	0.000
HOMA-IR ratio					
Unknown	8	0.511(-0.022,1.044)	0.060	82.7%	0.000
≤1.39	5	0.614(-0.370,1.599)	0.221	94.9%	0.000
1.40–1.53	5	0.626(-0.020,1.272)	0.057	85.8%	0.000
1.54–1.89	6	0.335(-0.354,1.023)	0.341	85.2%	0.000
>1.89	5	0.967(0.103,1.831)	0.028	93.5%	0.000
T ratio					
Unknown	7	0.695(-0.017,1.406)	0.056	84.5%	0.000
≤1.64	6	0.217(-0.104,0.537)	0.186	53.5%	0.056
1.65–1.81	5	0.258(-0.302,0.818)	0.366	86.4%	0.000
1.82–2.10	5	0.295(-0.290,0.881)	0.323	75.7%	0.002
>2.10	6	1.420(0.429,2.411)	0.005	96.1%	0.000

With regard to the subgroup analysis by ethnicity, significantly higher TNF-α levels were found in patients with PCOS in both Caucasian (SMD = 0.308, 95%CI = 0.005–0.612, P = 0.047, I^2^ = 79.8%) and Asia ethnicity (SMD = 1.103, 95%CI = 0.489–1.718, P = 0.000, I^2^ = 94.0%) (S1 Fig, Table 2). There were also significant differences in TNF-α levels of low-score studies (SMD = 0.360, 95%CI = 0.070–0.650, P = 0.015, I^2^ = 69.1%) and high-score studies (SMD = 0.840, 95%CI = 0.070–0.650, P = 0.003, I^2^ = 94.8%) (S2 Fig, Table 2).

The methods used to measure TNF-α levels included enzyme-linked immunosorbent assay(ELISA,), chemiluminescence(CL), radioimmunoassay(RIA), and multiplexing bead immunoassay(MBIA). The TNF-α levels were also significant higher in the ELISA group(SMD = 0.932, 95%CI = 0.361–1.502, P = 0.001, I^2^ = 94.1%), CL group(SMD = 0.284, 95%CI = 0.020–0.548, P = 0.035, I^2^ = 54.0%), and MBIA group(SMD = 1.059, 95%CI = 0.451–1.666, P = 0.001, I^2^ = 51.4%), but not in the RIA group (SMD = -0.255, 95%CI = -0.560–0.110, P = 0.188, I^2^ = 0.0%) (S3 Fig, Table 2).

Subgroup analyses also demonstrated higher TNF-α levels in subgroups with BMI of <25 kg/m^2^ and ≥25 kg/m^2^ (SMD = 0.829, 95% CI = 0.280–1.379, P = 0.003, I^2^ = 92.2% and SMD = 0.431, 95%CI = 0.024–0.838, P = 0.038, I^2^ = 89.3%, respectively; S4 Fig, Table 2).

In the subgroup analysis, significant difference in the TNF-α levels of PCOS patients versus the controls were observed in the subgroup with HOMA-IR ratio >1.72(SMD = 0.967, 95% CI = 0.103–1.831, P = 0.028, I^2^ = 93.5%)(S5 Fig, Table 2). However, there was no significant difference in the subgroups with unknown HOMA2-IR ratio, HOMA2-IR ratios ≤1.39, 1.40–1.53, and 1.54–1.89 (S5 Fig, Table 2). There was also a significant difference in the TNF-α levels in the subgroup with T ratio>2.10 (SMD = 1.420, 95% CI = 0.429–2.411, P = 0.005, I^2^ = 96.1%) but not in unknown T ratio group and low T ratio groups (T ratio ≤1.64, 1.65–1.81, and 1.82–2.10) (S6 Fig, Table 2).

#### Meta-regression analysis

To further investigate the sources of heterogeneity, meta-regression analysis was conducted. SMD was used as the dependent variable, and year, ethnicity, sample Size, quality score, BMI, Age, HOMA-IR ratio, and T ratio were entered as explanatory covariates. Results of the univariate analysis are presented in Table 3. If the regression coefficient of the covariates were significant at the level of 0.1, then the covariates were entered into the multivariate meta-regression. Based on the univariate meta-regression analysis, the regression coefficient of the ethnicity (Asia and Australia) was significant at the level of 0.1 (P = 0.069). Therefore, subsequent multivariate meta-regression could not be done. The results of meta-regression suggested that ethnicity might contribute little to the heterogeneity between included studies, and other covariates failed to account for the heterogeneity.

**Table 3:** Univariate meta-regression analysis for the potential variables between studies.

	Studies	Coefficient	SE	t	P	95%CI
Year	29	-0.008	0.043	-0.18	0.859	(0.096,0.080)
Ethnicity	29	-0.760	0.402	-1.89	0.069	(1.585,0.065)
Sample Size	29	0.001	0.003	0.45	0.653	(0.004,0.007)
Quality score	29	0.132	0.194	0.68	0.501	(0.266,0.532)
BMI	26	-0.049	0.039	-1.25	0.224	(0.130,0.032)
Age	25	-0.016	0.065	-0.24	0.812	(0.150,0.119)
HOMA-IR ratio	21	0.555	0.400	1.39	0.181	(0.282,1.392)
T ratio	22	0.472	0.304	1.55	0.136	(0.162,1.106)

#### Cumulative meta-analysis

**Fig 3:**
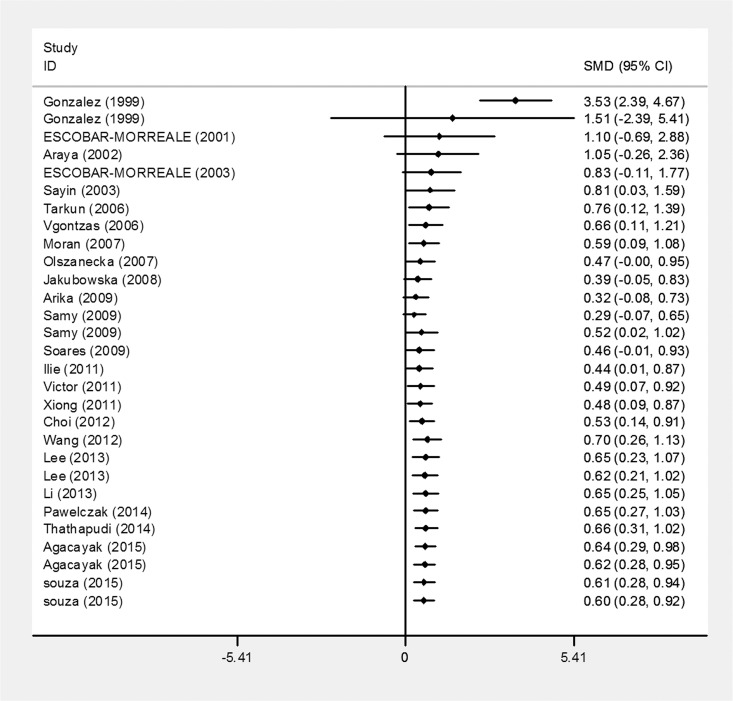
Cumulative meta-analysis for TNF-α levels in PCOS patients compared with controls.

To explore the changes in the TNF-α levels of PCOS patients over time, a cumulative meta-analysis was carried out (Fig 3). The random-effects pooled SMD was insignificantly larger or smaller than zero from the first study in 1999 to the study in 2009 by Soares et al., representing no statistically significant difference in TNF-α level between PCOS patients and healthy controls. A statistically significant difference was consistently observed since added the study by Ilie et al. (SMD = 0.44, 95% CI = 0.01–0.87), and the tendency of SMD became stable after that study, which indicating the stability of differences in the TNF-α levels of the PCOS patients over time.

#### Sensitivity analysis

**Fig 4:**
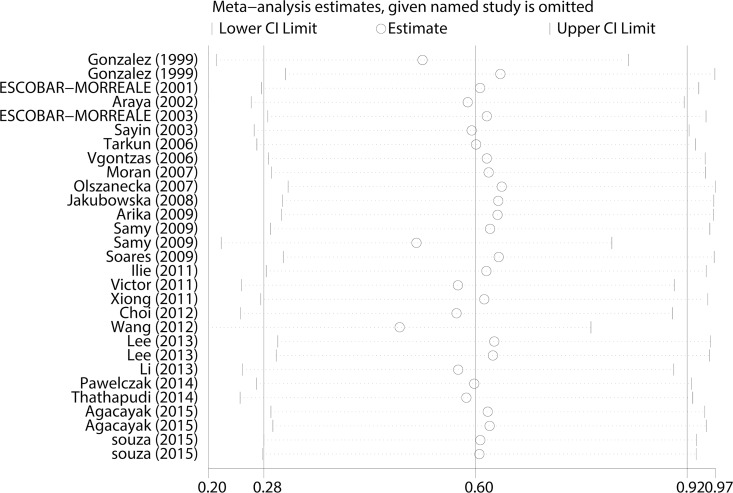
Sensitivity analysis for TNF-α levels in PCOS patients compared with controls.

A sensitivity analysis was performed by omitting one study at a time and calculating the pooled SMDs and 95% CIs for the remaining studies. There was no change in the effect when any one of the studies was excluded (Fig 4), which indicated that the results of the meta-analysis was reliable and stable. In three of included studies the BMI of the PCOS group and control group was unmatched. Therefore, the effect was recalculated after discarded these data in that three studies and there was also significant difference in the TNF-α levels of the PCOS patients compared with that of the controls (SMD = 0.589, 95% CI = 0.213–0.962, P = 0.002).

Studies that might contributed to the heterogeneity[11, 13, 15, 20–22, 24, 29, 61, 62], as confirmed by a Galbraith plot analysis (S7 Fig), were then excluded. The TNF-α levels remained significantly higher in the PCOS patients compared to the controls (SMD = 0.335, 95% CI = 0.162–0.508, P<0.001), but the heterogeneity decreased significantly (I2 = 25.7% and P = 0.159).

#### Publication bias

**Fig 5:**
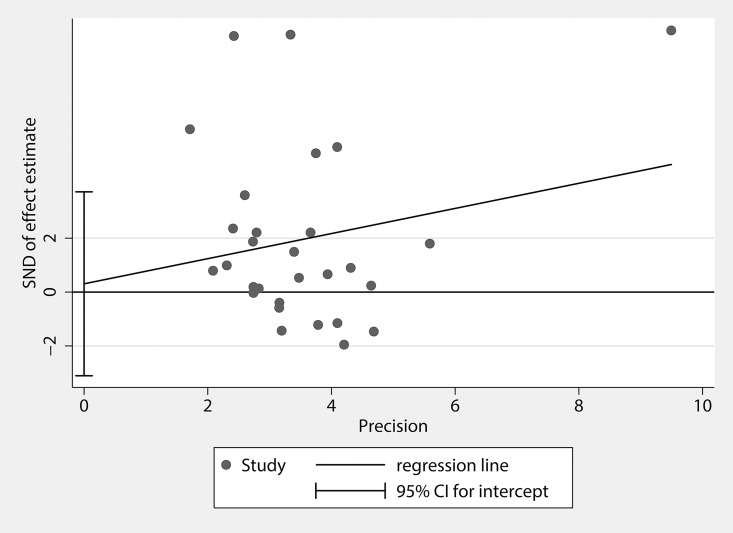
Graph of Egger’s test for publication bias in the studies of the meta-analysis of TNF-α levels in PCOS patients compared with controls.

Publication bias of the studies was evaluated by Begg’s funnel plots and Egger’s tests. The Begg’s funnel plot was slightly asymmetrical in distribution (P = 0.028), which indicated the possibility of publication bias. But the Egger’s test suggested no statistically significant asymmetry of the funnel plot (P = 0.855, Fig 5). We further undertook analysis using the trim-and-fill method[63]. The results from the trim-and-fill analysis did not change the summary estimate of effect, suggesting that publication bias is unlikely to affect the result of the meta-analysis.

## Discussion

In this meta-analysis with 29 included studies it was demonstrated that the TNF-α levels in PCOS patients were significantly higher than in controls, which were consistent with the results of toulis’s [31]. With regard to the subgroup analyses stratified by ethnicity and study quality, significantly high TNF-α levels were found in patients with PCOS in all of these subgroups. Furthermore, raised TNF-α levels were found both in lean and in obese PCOS patients. In the subgroup stratified by HOMA ratio and T ratio, significant difference was only observed in the quartiles with HOMA-IR ratio >1.72 and T ratio >2.10. So elevated TNF-α levels are related to the insulin resistance and androgen status in women with PCOS. By meta-regression it was suggested that ethnicity might contribute little to the heterogeneity between included studies. Through cumulative meta-analysis and sensitivity analysis it was concluded the difference in the TNF-α levels of the PCOS patients compared to the controls was stable and reliable.

Two meta-analyses of comparing circulating TNF-α levels in women with PCOS and healthy controls were conducted in 2011[30, 31]. The analysis by toulis et al[31] with 13 studies revealed that TNF-α levels were higher in women with PCOS than in controls, but the other meta-analysis with 9 studies conducted by Escobar-Morreale et al found there was no significant difference in TNF-α levels of PCOS women and controls[30]. In Escobar-Morreale’s meta-analysis four studies with fewer than 25 PCOS women were excluded, which resulted in the inconsistent effect with toulis’s study. So we conducted the analysis after discarding the seven studies with fewer than 25 PCOS patients[8, 9, 14, 16, 19, 26, 28], the effect direction (SMD = 0.594, 95% CI = 0.192–0.996, P = 0.004) was the same as the analysis with 29 studies. This study included 29 studies and the sample size was twice more than that of the previous studies. With the expansion of sample size, the corresponding statistical power can be increased. The BMI of the cases and controls was unmatched in three of included studies. Considering that the levels of proinflammatory cytokines are usually elevated in obesity [64], potential selection bias could influence the results in BMI-unmatched studies. So the effect was recalculated after excluding these data and the result was also significant different in the TNF-α levels.

In our study TNF-α levels of PCOS patients were higher than those of the controls in the subgroups with high HOMA-IR ratio and T ratio, but no significant difference in the low HOMA2-IR ratio and T ratio subgroups. This indicates that the elevated TNF-α levels are directly related to the insulin resistance and androgen excess of PCOS. TNF-α levels were elevated in both obesity and metabolic syndrome[65]. It decreased the cellular response to insulin in adipocytes, hepatocytes and human muscle cells [64].In Gonzalez’s study women with PCOS failed to suppress monocyte-derived TNF-α and IL-6 release in response to glucose ingestion, and this response is independent of excess adiposity[56]. Monocyte-derived cytokine release was directly related to insulin resistance and androgen excess[56]. Combined with our study it was supposed that the raised TNF-α levels are not an intrinsic characteristic of PCOS, but it may be involved in promoting insulin resistance and androgen excess of PCOS. The mechanism of TNF-α to participate in insulin resistance and androgen excess could be of potential research interest in PCOS.

To our knowledge, this is the most comprehensive meta-analysis uptodate to evaluate the association between TNF-α levels and PCOS. Substantial number of cases and controls were pooled from publications concerned with circulating TNF-α levels and PCOS, which greatly increased statistical power of the analysis and provided enough evidence to draw a safe conclusion. Sensitivity analysis suggested that no single study influenced the pooled SMD qualitatively. And cumulative meta-analysis showed that no significant change occurred in pooled SMD since 2009, indicating the stability of the association between high TNF-α levels and PCOS. In addition, no obvious publication bias was detected in this meta-analysis, which indicated that the pooled results of our study should be reliable. Taken together, these data further confirm the reliability and stability of the meta-analysis results.

The main limitation of the present study is the significant heterogeneity across the included studies. After stratified by methods to measure TNF-α levels, it is found that the heterogeneity was decreased in the CL, RIA, and MBIA group, but also high in the ELISA group. So the heterogeneity in this meta-analysis might be partly attributed to the differences on the sensitivity and accuracy of different methods used to measure TNF-α levels. In the subgroup of T ratio ≤1.64 this heterogeneity decreased to 53.5%. So the differences in T levels may also be related to the heterogeneity. By meta-regression only ethnicity was found to contribute little to the heterogeneity. According to Galbraith plot analysis ten studies that might contributed to the heterogeneity were found. After excluding these studies the TNF-α levels remained significantly high in the PCOS patients, but the heterogeneity decreased to 25.7%. Although we conducted subgroup analysis, meta-regression analysis, and sensitivity analysis, the sources of heterogeneity were not satisfactorily explained. It might reflect clinical heterogeneity related to geographical differences, smoking status, concomitant subclinical inflammatory diseases, and variability in PCOS diagnostic criteria. But it was not possible to take into account different PCOS phenotypes.

Although the publication bias was denied by egger’s test and trim-and-fill method, the possibility of undetected bias cannot be absolutely excluded. In addition, missing data on the HOMA-IR ratio and T ratio in some studies may produce a certain degree of systemic bias. The results were based on unadjusted estimates, whereas a more precise evaluation should consider the confounding factors such as smoking status, alcoholic consumption, environmental factors, and other diet lifestyle. There was an increase of TNF-α in patients with dyslipidemia [64], but because of data limitation we were not able to conduct further analysis stratified by the lipid metabolism of included women. These limitations must be considered when interpreting the results of this meta-analysis.

## Conclusions

This meta-analysis suggested that circulating TNF-α levels in women with PCOS were significantly higher than those in healthy controls and that a high serum TNF-α concentration was related to insulin resistance and androgen excess but not to the BMI. Therefore, a high TNF-α level is not an intrinsic characteristic of PCOS, but it may be involved in promoting insulin resistance and hyperandrogenism of PCOS.

## Supporting Information

S1 Fig The quantitative synthesis for TNF-α levels in PCOS patients compared with controls stratified by Ethnicity. (TIF)

S2 Fig The quantitative synthesis for TNF-α levels in PCOS patients compared with controls stratified by Study quality. (TIF)

S3 Fig The quantitative synthesis for TNF-α levels in PCOS patients compared with controls stratified by methods used to measure TNF-α levels. (TIF)

S4 Fig The quantitative synthesis for TNF-α levels in PCOS patients compared with controls stratified by BMI. (TIF)

S5 Fig The quantitative synthesis for TNF-α levels in PCOS patients compared with controls stratified by HOMA-IR ratio. (TIF)

S6 Fig The quantitative synthesis for TNF-α levels in PCOS patients compared with controls stratified by T ratio. (TIF)

S7 Fig Galbraith plots of TNF-α levels in PCOS patients compared with controls. (TIF)

S1 File Search Strategies. (DOC)

S2 File Excluded articles with reasons. (DOCX)

S1 Table Quality Assessment of Included Studies. (DOCX)

S2 Table The data of included studies in the meta-analysis. (DOCX)
