# Supplementary figures and images for: High Serum Tumor Necrosis Factor-Alpha Levels in Women with Polycystic Ovary Syndrome: A Meta-Analysis
Source: PLoS One. 2016 Oct 20;11(10):e0164021. doi: 10.1371/journal.pone.0164021 (PMC5072730; doi:10.1371/journal.pone.0164021)

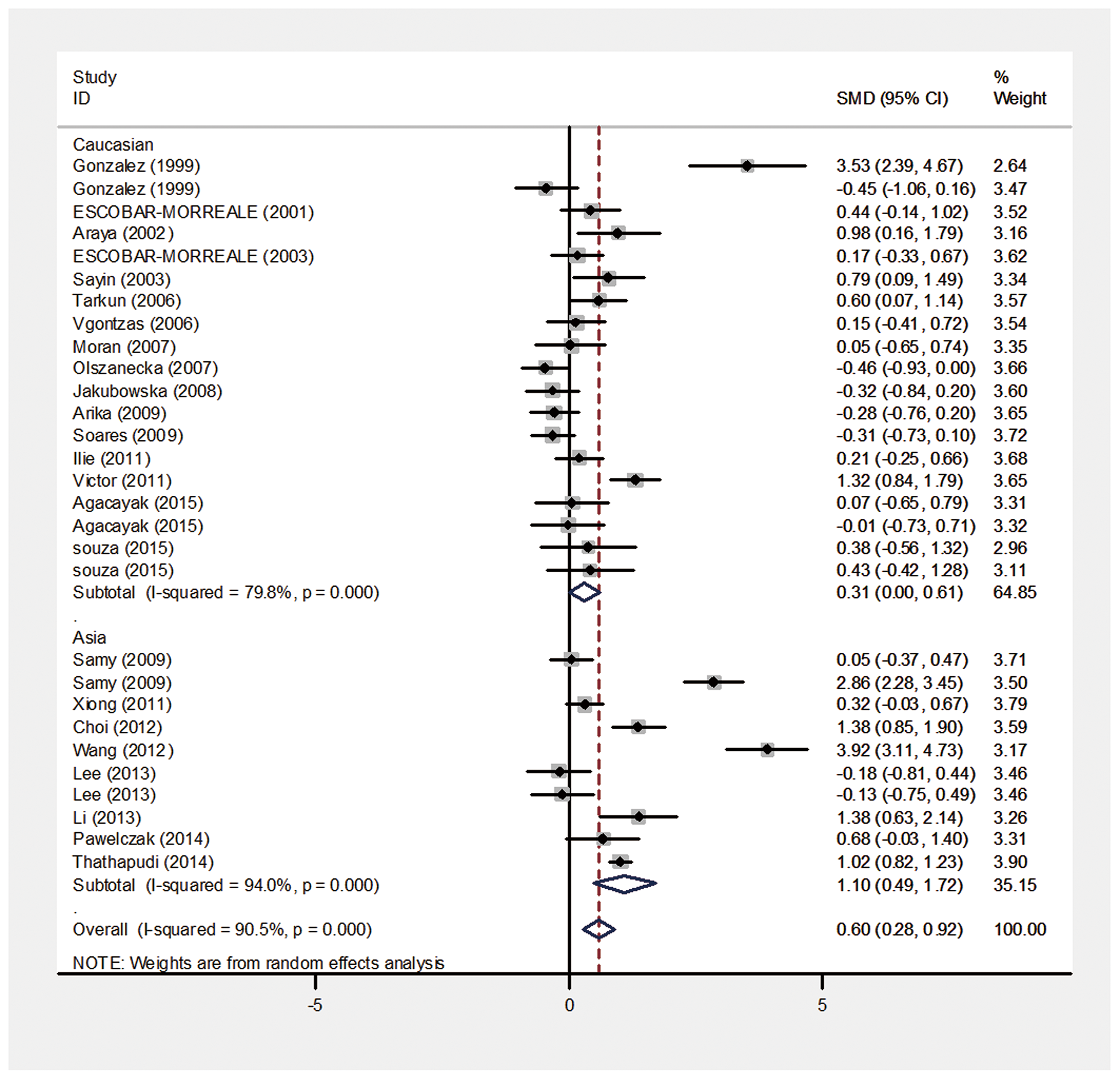

Supplement: S1 Fig — (TIF) [file pone.0164021.s001.tif]

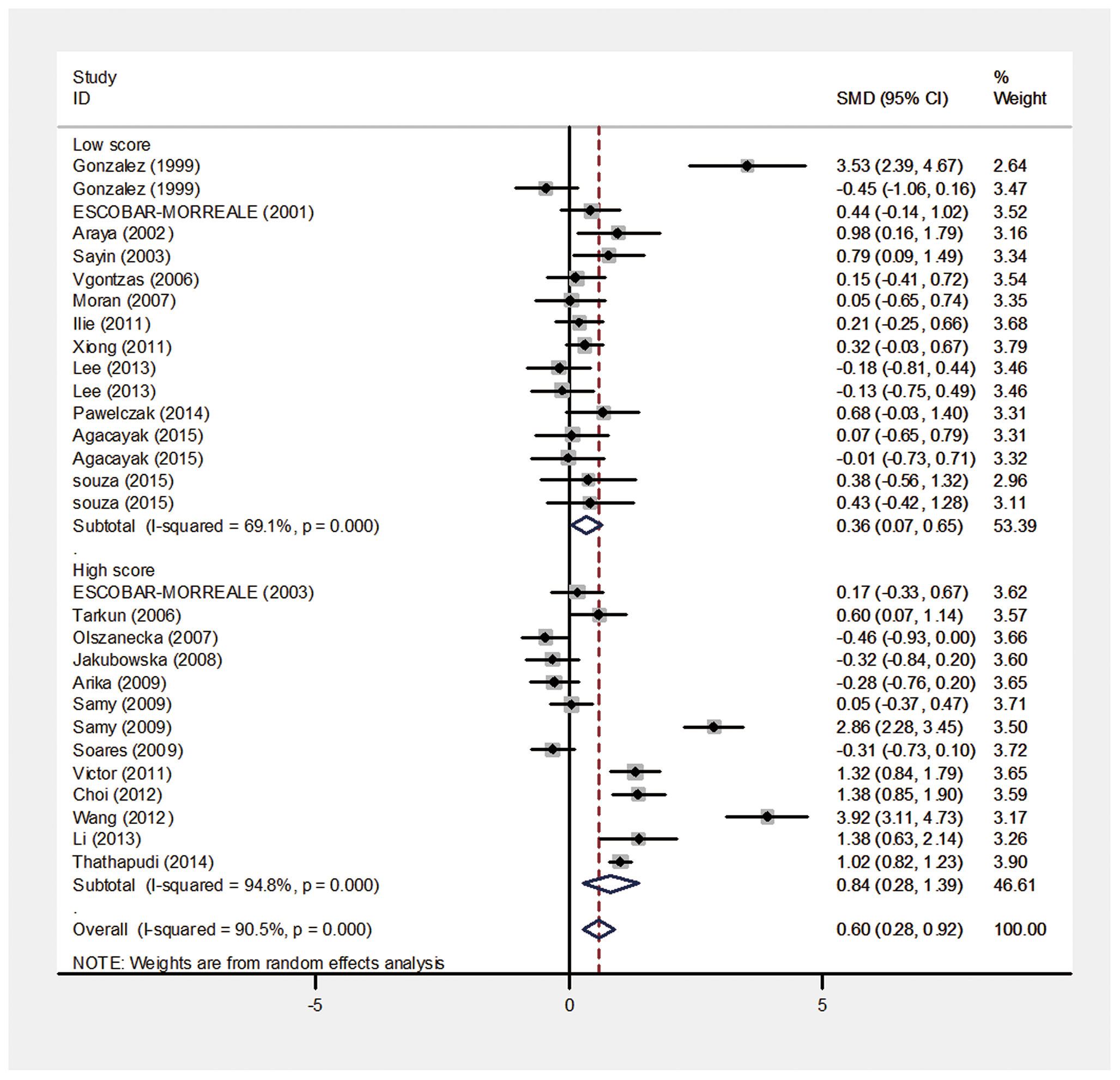

Supplement: S2 Fig — (TIF) [file pone.0164021.s002.tif]

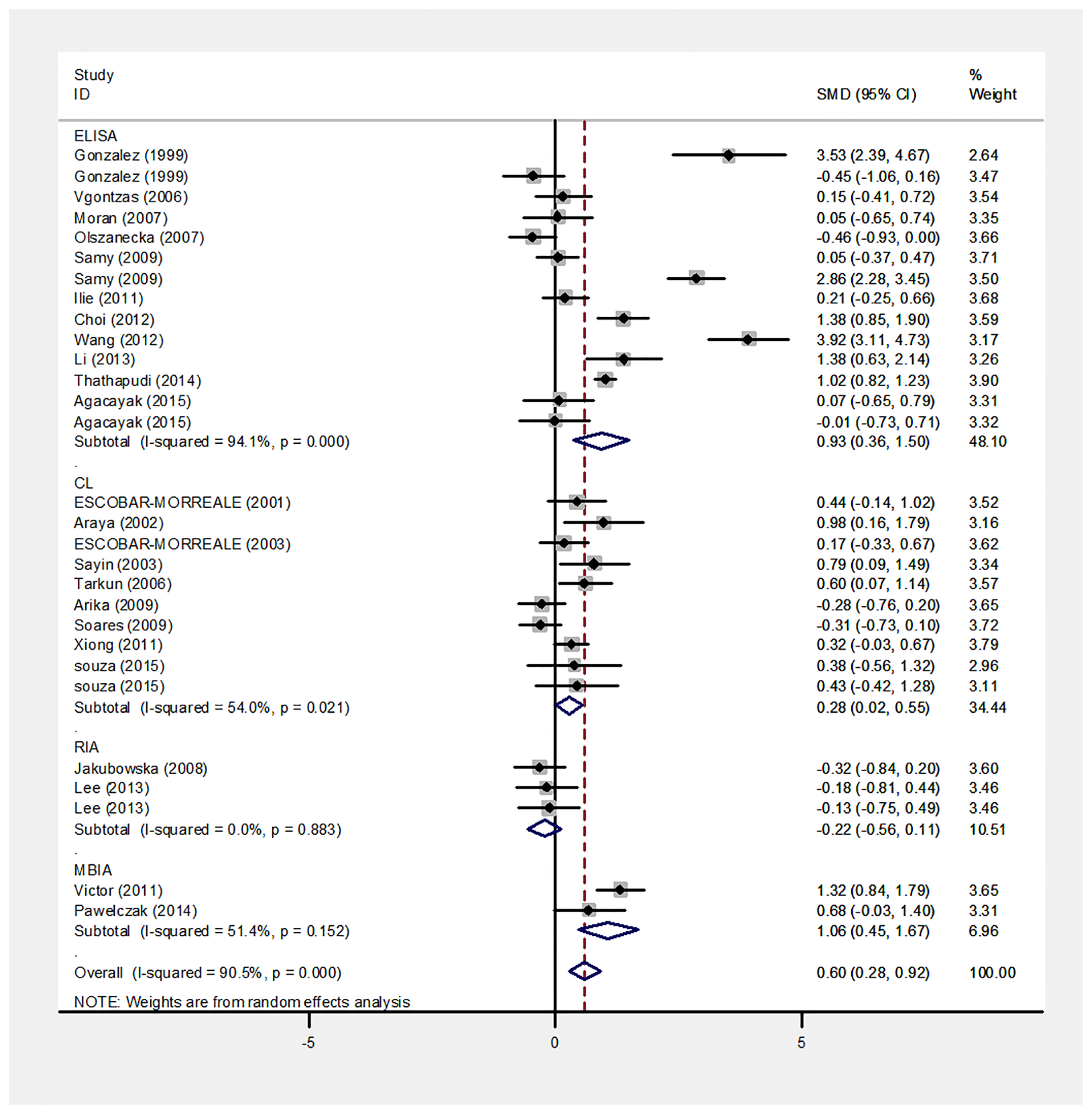

Supplement: S3 Fig — (TIF) [file pone.0164021.s003.tif]

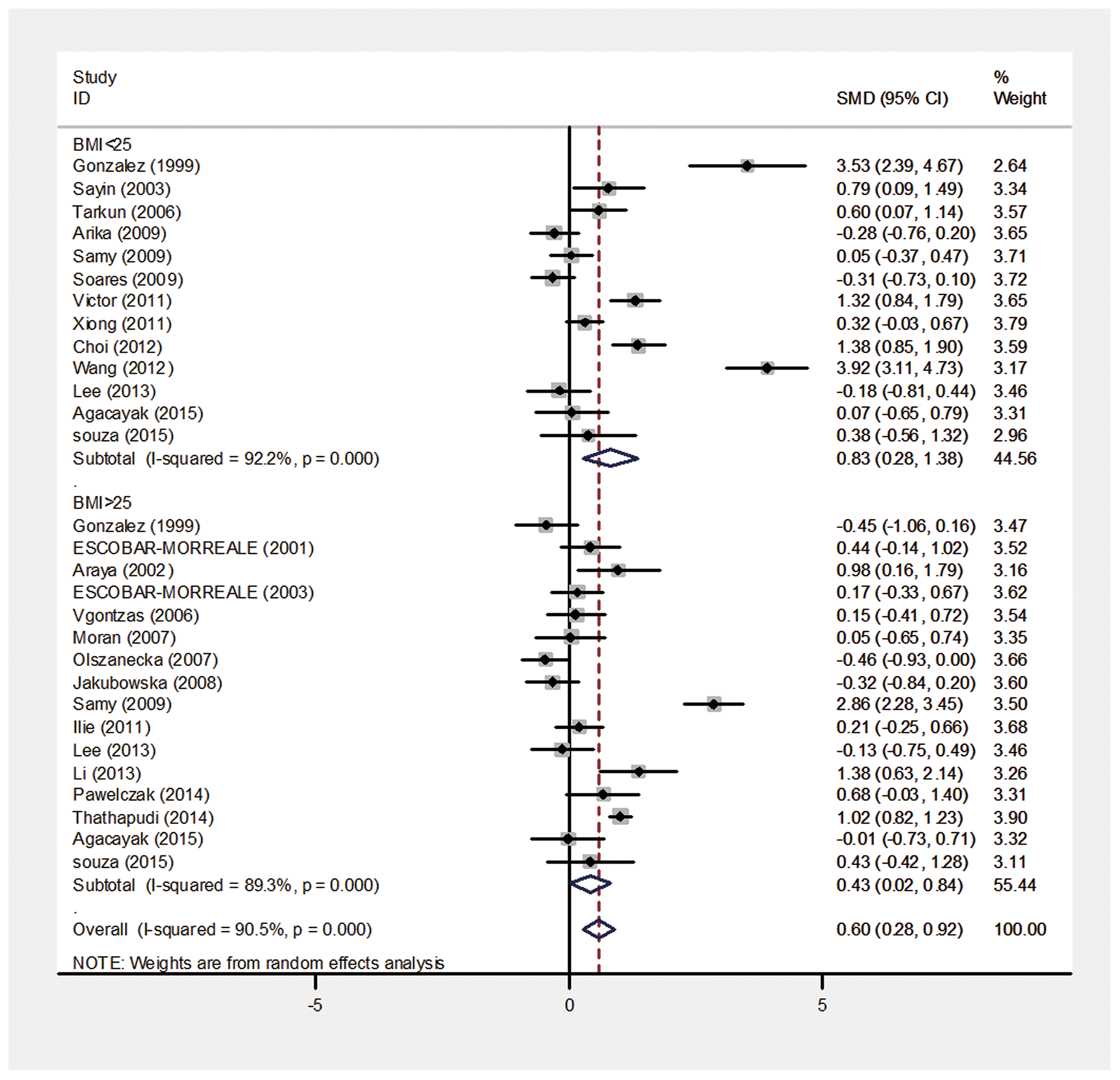

Supplement: S4 Fig — (TIF) [file pone.0164021.s004.tif]

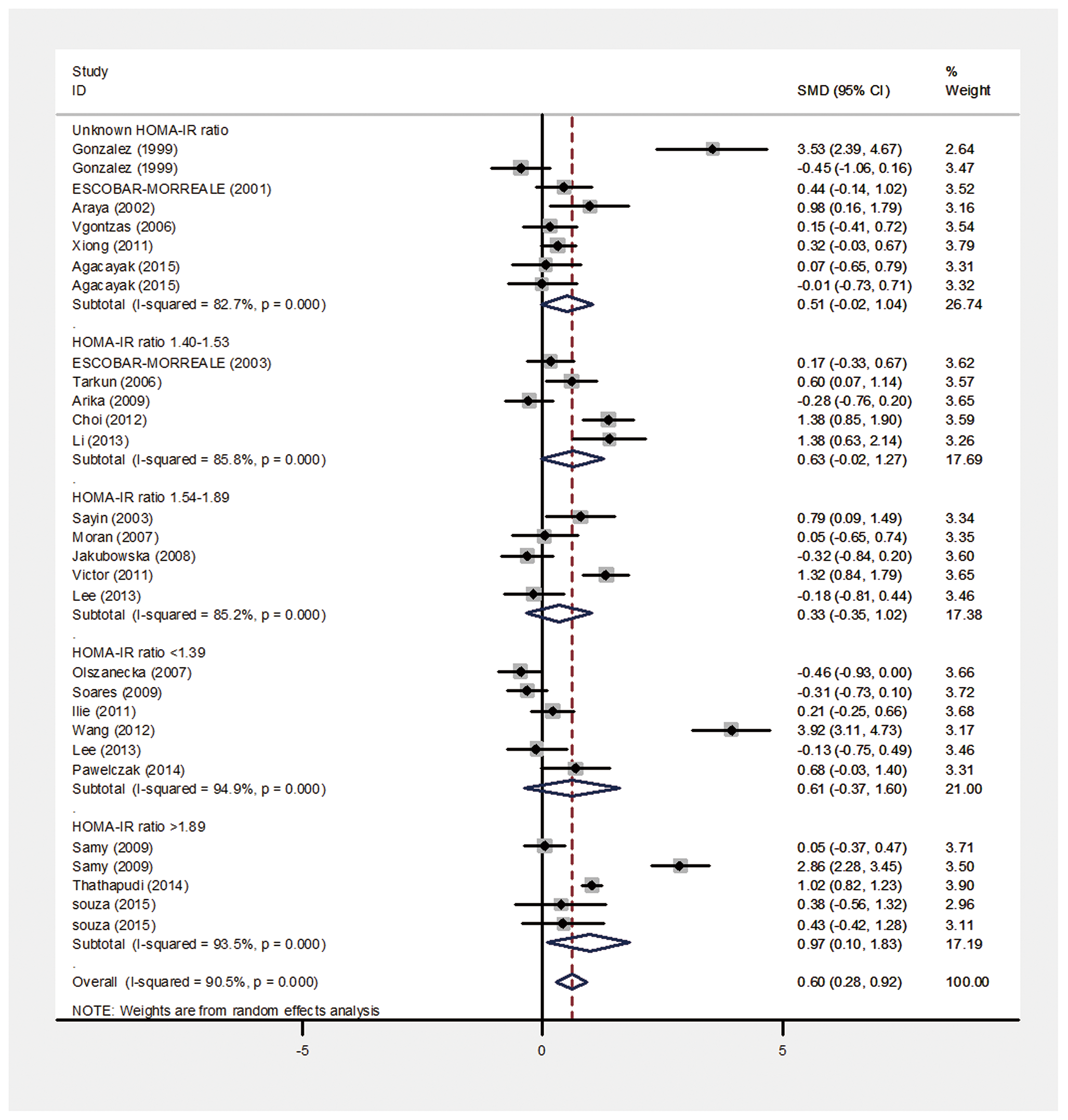

Supplement: S5 Fig — (TIF) [file pone.0164021.s005.tif]

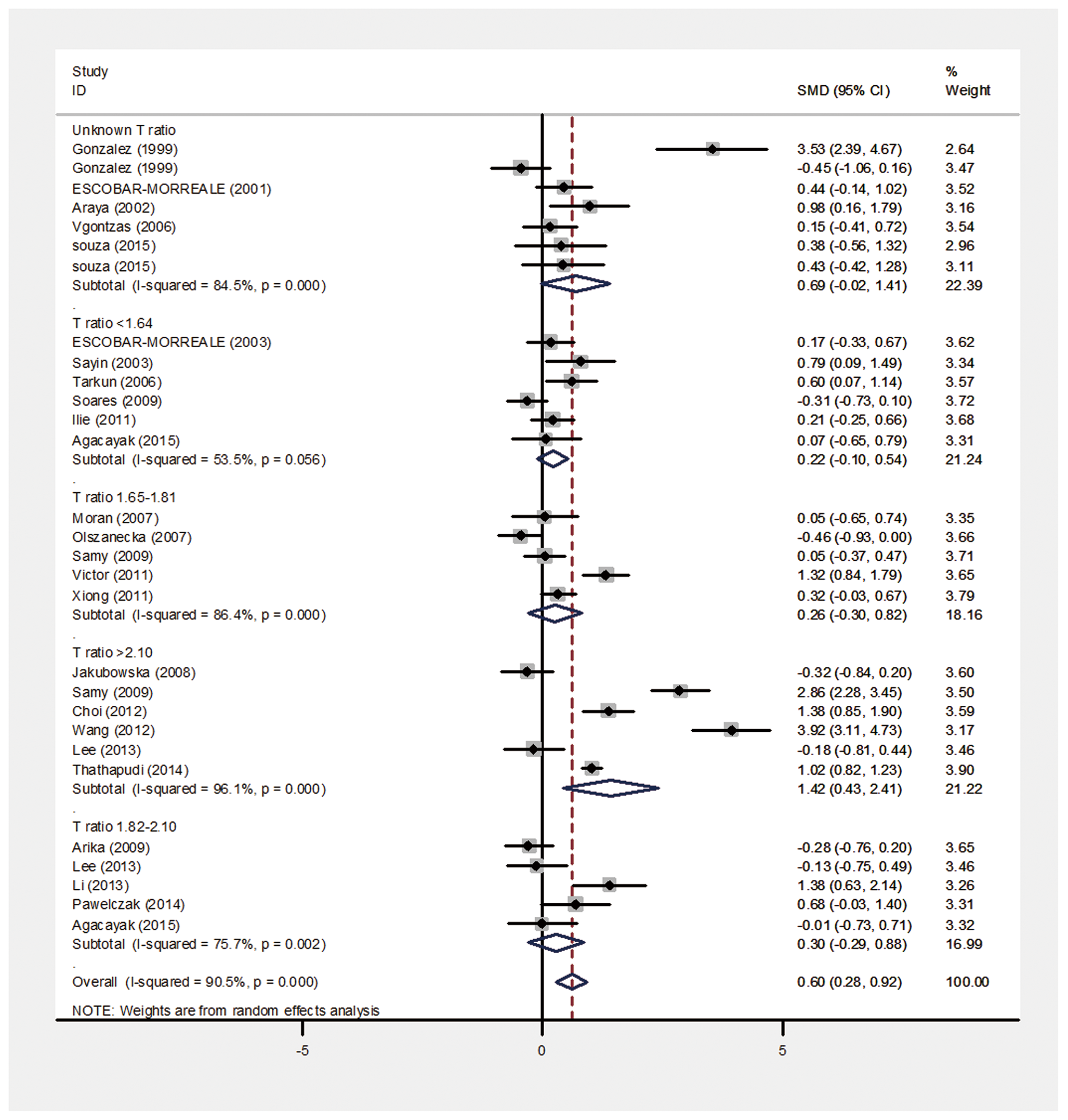

Supplement: S6 Fig — (TIF) [file pone.0164021.s006.tif]

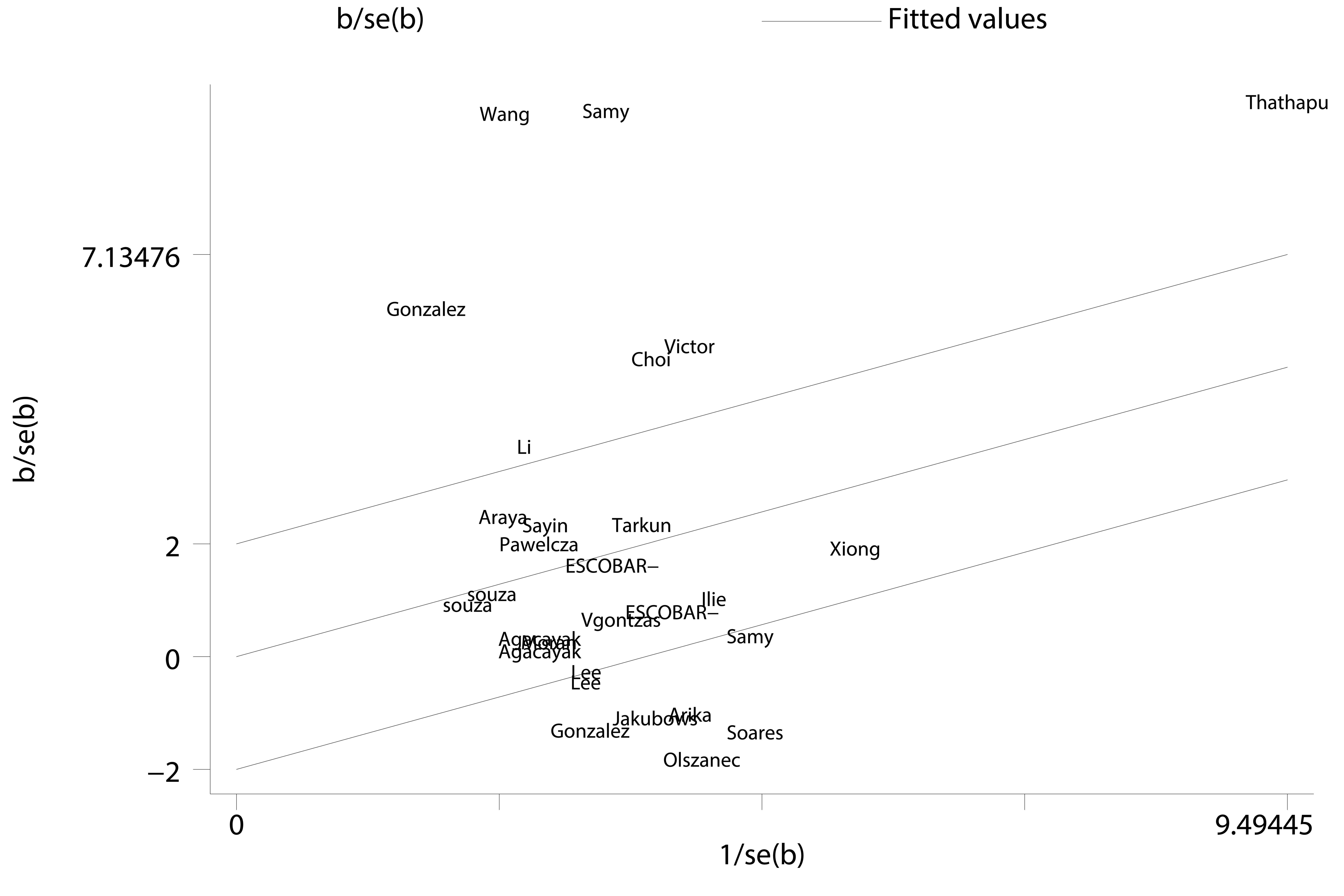

Supplement: S7 Fig — (TIF) [file pone.0164021.s007.tif]
