# Supplementary material for: High Serum Tumor Necrosis Factor-Alpha Levels in Women with Polycystic Ovary Syndrome: A Meta-Analysis
Source: PLoS One. 2016 Oct 20;11(10):e0164021. doi: 10.1371/journal.pone.0164021 (PMC5072730; doi:10.1371/journal.pone.0164021)
Supplement: S1 File — (DOC) [file pone.0164021.s008.doc]

Search Strategies

**Medline and Embase (Embase.com)**

| ('ovary polycystic disease'/exp OR (polycystic NEAR/5 ovar*):de,ab,ti OR pco*:de,ab,ti OR leventhal:de,ab,ti OR (micropolycystic NEAR/5 ovar*):de,ab,ti OR (sclerocystic NEAR/5 ovar*):de,ab,ti) AND ('tumor necrosis factor alpha'/exp OR 'tum*r necrosis factor-al*a':de,ab,ti OR 'TNF-al*a':de,ab,ti OR 'tum*r necrosis factor-α':de,ab,ti OR 'TNF-α':de,ab,ti)  **Cochrane library**  #1 MeSH descriptor: [Polycystic Ovary Syndrome] explode all trees  #2 polycystic near/5 ovar* or pco* or leventhal or micropolycystic near/5 ovar* or sclerocystic near/5 ovar* |
| --- |

#3 #1 OR #2

#4 MeSH descriptor: [Tumor Necrosis Factor-alpha] explode all trees

#5 tum*r necrosis factor-al*a OR TNF-al*a OR tum*r necrosis factor-α OR TNF-α

#6 #4 or #5

#7 #3 AND #6
