# Supplementary material for: High Serum Tumor Necrosis Factor-Alpha Levels in Women with Polycystic Ovary Syndrome: A Meta-Analysis
Source: PLoS One. 2016 Oct 20;11(10):e0164021. doi: 10.1371/journal.pone.0164021 (PMC5072730; doi:10.1371/journal.pone.0164021)
Supplement: S2 File — (DOCX) [file pone.0164021.s009.DOCX]

Excluded artilces with reasons:

Data not extractable：1-13

Inappropriate control：14-21

mRNA levels：25-27

Not from blood sample：22-24

Sample overlap: 28

1. Amato G, Conte M, Mazziotti G, Lalli E, Vitolo G, Tucker AT, et al. Serum and follicular fluid cytokines in polycystic ovary syndrome during stimulated cycles. Obstet Gynecol. 2003;101(6):1177-82. doi: 10.1016/s0029-7844(03)00233-3. PubMed PMID: WOS:000183293300009.

2. Puder JJ, Varga S, Nusbaumer CPG, Zulewski H, Bilz S, Muller B, et al. Women with polycystic ovary syndrome are sensitive to the TNF-alpha-lowering effect of glucose-induced hyperinsulinaemia. European Journal of Clinical Investigation. 2006;36(12):883-9. doi: 10.1111/j.1365-2362.2006.01734.x. PubMed PMID: WOS:000241832700007.

3. Shroff R, Kerchner A, Maifeld M, Van Beek EJR, Jagasia D, Dokras A. Young obese women with polycystic ovary syndrome have evidence of early coronary atherosclerosis. Journal of Clinical Endocrinology & Metabolism. 2007;92(12):4609-14. doi: 10.1210/jc.2007-1343. PubMed PMID: WOS:000251399700019.

4. Ozcaka O, Ceyhan BO, Akcali A, Bicakci N, Lappin DF, Buduneli N. Is There an Interaction Between Polycystic Ovary Syndrome and Gingival Inflammation? Journal of Periodontology. 2012;83(12):1529-37. doi: 10.1902/jop.2012.110588. PubMed PMID: WOS:000312355600012.

5. Svendsen PF, Christiansen M, Hedley PL, Nilas L, Pedersen SB, Madsbad S. Adipose expression of adipocytokines in women with polycystic ovary syndrome. Fertility and Sterility. 2012;98(1). doi: 10.1016/j.fertnstert.2012.03.056. PubMed PMID: WOS:000305950200046.

6. Barcellos CRG, Rocha MP, Hayashida SAY, Dantas WS, Yance VRV, Marcondes JAM. Obesity, but not polycystic ovary syndrome, affects circulating markers of low-grade inflammation in young women without major cardiovascular risk factors. Hormones. 2015;14(2):251-7.

7. Grimaldi Barcellos CR, Rocha MP, Yamashita Hayashida SA, Dantas WS, Vieira Yance VdR, Miguel Marcondes JA. Obesity, but not polycystic ovary syndrome, affects circulating markers of low-grade inflammation in young women without major cardiovascular risk factors. Hormones-International Journal of Endocrinology and Metabolism. 2015;14(2):251-7. PubMed PMID: WOS:000358629900008.

8. Puder JJ, Varga S, Kraenzlin M, De Geyter C, Keller U, Muller B. Central fat excess in polycystic ovary syndrome: Relation to low-grade inflammation and insulin resistance. Journal of Clinical Endocrinology & Metabolism. 2005;90(11):6014-21. doi: 10.1210/jc.2005-1002. PubMed PMID: WOS:000233115700014.

9. Gao H, Meng J, Xu M, Zhang S, Ghose B, Liu J, et al. Serum Heat Shock Protein 70 Concentration in Relation to Polycystic Ovary Syndrome in a Non-Obese Chinese Population. Plos One. 2013;8(6). doi: 10.1371/journal.pone.0067727. PubMed PMID: WOS:000320576400192.

10. Thomann R, Rossinelli N, Keller U, Tirri BF, De Geyter C, Ruiz J, et al. Differences in low-grade chronic inflammation and insulin resistance in women with previous gestational diabetes mellitus and women with polycystic ovary syndrome. Gynecological endocrinology : the official journal of the International Society of Gynecological Endocrinology. 2008;24(4):199-206. doi: 10.1080/09513590801893398. PubMed PMID: 18382906.

11. Knebel B, Janssen OE, Hahn S, Jacob S, Gleich J, Kotzka J, et al. Increased low grade inflammatory serum markers in patients with polycystic ovary syndrome (PCOS) and their relationship to PPAR gamma gene variants. Experimental and Clinical Endocrinology & Diabetes. 2008;116(8):481-6. doi: 10.1055/s-2008-1058085. PubMed PMID: WOS:000259927300005.

12. Gonzalez F, Sia CL, Shepard MK, Rote NS, Minium J. Inflammation in Response to Glucose Ingestion Is Independent of Excess Abdominal Adiposity in Normal-Weight Women with Polycystic Ovary Syndrome. Journal of Clinical Endocrinology & Metabolism. 2012;97(11):4071-9. doi: 10.1210/jc.2012-2131. PubMed PMID: WOS:000310710500056.

13. Victor VM, Rovira-Llopis S, Bañuls C, Diaz-Morales N, Lopez-Domenech S, Escribano-López I, et al. Metformin modulates human leukocyte/endothelial cell interactions and proinflammatory cytokines in polycystic ovary syndrome patients. Atherosclerosis. 2015;242(1):167-73.

14. Kawamura S, Maesawa C, Nakamura K, Nakayama K, Morita M, Hiruma Y, et al. Predisposition for borderline personality disorder with comorbid major depression is associated with that for polycystic ovary syndrome in female Japanese population. Neuropsychiatr Dis Treat. 2011;7:655-62. doi: 10.2147/ndt.s25504. PubMed PMID: WOS:000297997100001.

15. Oner G, Muderris II. Clinical, endocrine and metabolic effects of metformin vs N-acetyl-cysteine in women with polycystic ovary syndrome. European Journal of Obstetrics & Gynecology and Reproductive Biology. 2011;159(1):127-31. doi: 10.1016/j.ejogrb.2011.07.005. PubMed PMID: WOS:000298202800024.

16. Oner G, Muderris II. Efficacy of omega-3 in the treatment of polycystic ovary syndrome. Journal of Obstetrics and Gynaecology. 2013;33(3):289-91.

17. Almario RU, Karakas SE. Roles of Circulating WNT-Signaling Proteins and WNT-Inhibitors in Human Adiposity, Insulin Resistance, Insulin Secretion, and Inflammation. Hormone and Metabolic Research. 2015;47(2):152-7. doi: 10.1055/s-0034-1384521. PubMed PMID: WOS:000350739000010.

18. Gower BA, Goss AM. A Lower-Carbohydrate, Higher-Fat Diet Reduces Abdominal and Intermuscular Fat and Increases Insulin Sensitivity in Adults at Risk of Type 2 Diabetes. Journal of Nutrition. 2015;145(1):177-83. doi: 10.3945/jn.114.195065. PubMed PMID: WOS:000347263600026.

19. Covington JD, Tam CS, Pasarica M, Redman LM. Higher circulating leukocytes in women with PCOS is reversed by aerobic exercise. Biochimie. 2016;124:27-33.

20. Olszanecka-Glinianowicz M, Zahorska-Markiewicz B, Kocelak P, Janowska J, Semik-Grabarczyk E. The effect of weight loss on inflammation in obese women with polycystic ovary syndrome. Endokrynologia Polska. 2008;59(1):13-7. PubMed PMID: 18335395.

21. Omu AE, Al-Azemi MK, Makhseed M, Al-Oattan F, Ismail AA, Al-Tahir S, et al. Differential expression of T-helper cytokines in the peritoneal fluid of women with normal ovarian cycle compared with women with chronic anovulation. Acta obstetricia et gynecologica Scandinavica. 2003;82(7):603-9. PubMed PMID: 12790840.

22. Victor VM, Rocha M, Banuls C, Sanchez-Serrano M, Sola E, Gomez M, et al. Mitochondrial Complex I Impairment in Leukocytes from Polycystic Ovary Syndrome Patients with Insulin Resistance. Journal of Clinical Endocrinology & Metabolism. 2009;94(9):3505-12. doi: 10.1210/jc.2009-0466. PubMed PMID: WOS:000269584600050.

23. Gonzalez F, Kirwan JP, Rote NS, Minium J. Evidence of mononuclear cell preactivation in the fasting state in polycystic ovary syndrome. American Journal of Obstetrics and Gynecology. 2014;211(6). doi: 10.1016/j.ajog.2014.06.044. PubMed PMID: WOS:000346585300015.

24. Gonzalez F, Sia CL, Shepard MK, Rote NS, Minium J. The Altered Mononuclear Cell-Derived Cytokine Response to Glucose Ingestion Is Not Regulated by Excess Adiposity in Polycystic Ovary Syndrome. Journal of Clinical Endocrinology & Metabolism. 2014;99(11):E2244-E51. doi: 10.1210/jc.2014-2046. PubMed PMID: WOS:000346743100066.

25. Lindholm A, Blomquist C, Bixo M, Dahlbom I, Hansson T, Poromaa IS, et al. No difference in markers of adipose tissue inflammation between overweight women with polycystic ovary syndrome and weight-matched controls. Human Reproduction. 2011;26(6):1478-85. doi: 10.1093/humrep/der096. PubMed PMID: WOS:000290818400024.

26. Seow K-M, Lin Y-H, Hwang J-L, Wang P-H, Ho L-T, Lin Y-H, et al. Expression levels of haem oxygenase-I in the omental adipose tissue and peripheral blood mononuclear cells of women with polycystic ovary syndrome. Human Reproduction. 2011;26(2):431-7. doi: 10.1093/humrep/deq351. PubMed PMID: WOS:000286470500021.

27. Huang ZH, Manickam B, Ryvkin V, Zhou XJ, Fantuzzi G, Mazzone T, et al. PCOS Is Associated with Increased CD11c Expression and Crown-Like Structures in Adipose Tissue and Increased Central Abdominal Fat Depots Independent of Obesity. Journal of Clinical Endocrinology & Metabolism. 2013;98(1):E17-E24. doi: 10.1210/jc.2012-2697. PubMed PMID: WOS:000316210300003.

28. Tao T, Li S, Zhao A, Zhang Y, Liu W. Expression of the CD11c gene in subcutaneous adipose tissue is associated with cytokine level and insulin resistance in women with polycystic ovary syndrome. European Journal of Endocrinology. 2012;167(5):705-13. doi: 10.1530/eje-12-0340. PubMed PMID: WOS:000309809400013.
