# Supplementary material for: High Serum Tumor Necrosis Factor-Alpha Levels in Women with Polycystic Ovary Syndrome: A Meta-Analysis
Source: PLoS One. 2016 Oct 20;11(10):e0164021. doi: 10.1371/journal.pone.0164021 (PMC5072730; doi:10.1371/journal.pone.0164021)
Supplement: S1 Table — (DOCX) [file pone.0164021.s010.docx]

**S1 Table. Quality Assessment of Included Studies**

| study | Case defined with independent validation | Representativeness of the cases | Controls from the same community | Controls have no history of disease | matched by BMI or age | matched by additional factor | sample size >50 | Overall score |
| --- | --- | --- | --- | --- | --- | --- | --- | --- |
| Gonzalez | 1 | 1 | 0 | 1 | 1 | 0 | 1 | 5 |
| ESCOBAR-MORREALE | 1 | 1 | 0 | 1 | 1 | 0 | 0 | 4 |
| Araya | 1 | 1 | 0 | 1 | 1 | 0 | 0 | 4 |
| ESCOBAR-MORREALE | 1 | 1 | 0 | 1 | 1 | 1 | 1 | 6 |
| Sayin | 1 | 0 | 0 | 1 | 1 | 0 | 0 | 3 |
| Tarkun | 1 | 1 | 0 | 1 | 1 | 1 | 1 | 6 |
| Vgontzas | 1 | 0 | 1 | 1 | 1 | 0 | 1 | 5 |
| Moran | 1 | 1 | 1 | 0 | 1 | 1 | 0 | 5 |
| Olszanecka | 1 | 1 | 0 | 1 | 1 | 1 | 1 | 6 |
| Jakubowska | 1 | 1 | 0 | 1 | 1 | 1 | 1 | 6 |
| Arikan | 1 | 1 | 0 | 1 | 1 | 1 | 1 | 6 |
| Samy | 1 | 1 | 0 | 1 | 1 | 1 | 1 | 6 |
| Soares | 1 | 1 | 0 | 1 | 1 | 1 | 1 | 6 |
| Ilie | 1 | 1 | 0 | 1 | 1 | 0 | 1 | 5 |
| Victor | 1 | 1 | 0 | 1 | 1 | 1 | 1 | 6 |
| Xiong | 1 | 0 | 0 | 0 | 1 | 0 | 1 | 3 |
| Choi | 1 | 1 | 1 | 1 | 1 | 1 | 1 | 7 |
| Wang | 1 | 1 | 0 | 1 | 1 | 1 | 1 | 6 |
| Lee | 1 | 1 | 0 | 1 | 1 | 0 | 1 | 5 |
| Li | 1 | 1 | 1 | 1 | 1 | 1 | 0 | 6 |
| Pawelczak | 1 | 0 | 0 | 1 | 1 | 0 | 0 | 3 |
| Thathapudi | 1 | 1 | 1 | 1 | 1 | 0 | 1 | 6 |
| Agacayak | 1 | 1 | 0 | 1 | 1 | 0 | 1 | 5 |
| souza | 1 | 1 | 0 | 1 | 1 | 0 | 0 | 4 |

The quality assessment criteria used in the study were: 1. whether the diagnosis of the PCOS was with independent validation; 2. whether the involved cases were representative of population; 3. whether the controls enrolled were from the same community; 4. whether the controls were described to have no history of disease; 5. whether the cases and controls were matched for age or BMI ; 6. Whether the cases and controls were matched for additional factor, such as drinking or smoking status; 7. whether the test methods were the same for cases and controls; 9. whether the sample size was >50. For each criterion a score of 0 or 1 was assigned according to whether the criterion was satisfactorily fulfilled. According to the quality score assessment, the distribution of the scores was between 0 and 6. Studies with score of 5 or above are classified as high quality studies. Others are categorized as low quality studies.
