# Supplementary material for: High Serum Tumor Necrosis Factor-Alpha Levels in Women with Polycystic Ovary Syndrome: A Meta-Analysis
Source: PLoS One. 2016 Oct 20;11(10):e0164021. doi: 10.1371/journal.pone.0164021 (PMC5072730; doi:10.1371/journal.pone.0164021)
Supplement: S2 Table — (DOCX) [file pone.0164021.s011.docx]

**S2 Table. The data of included studies in the meta-analysis**

| **Study** | **Ethicinity** | **Sample size** | **Score** | **BMI(kg/m^2^)** | |  | **Age(year)** | | **HOMA-IR ratio** | **T-ratio** |
| --- | --- | --- | --- | --- | --- | --- | --- | --- | --- | --- |
|  |  |  |  | **PCOS** | **Control** |  | **PCOS** | **Control** |  |  |
| Gonzalez | Caucasian | 32 | 5 | 24.2 ± 0.7 | 20.8 ± 0.8 |  | - | - | - | - |
| Gonzalez | Caucasian | 42 | 5 | 36.6 ±1.2 | 36.7 + 1.6 |  | - | - | - | - |
| Escobar-morreale | Caucasian | 48 | 4 | 29.0 ± 8.4 | 28.9 ± 7.7 |  | 24.3 ± 6.6 | 30.2 ± 8.7 | - | - |
| Araya | Caucasian | 27 | 4 | 32.7 ± 5.7 | 23 ± 2.2 |  | 25.6 ± 4.5 | 26.7 ± 4 | - | - |
| Escobar-morreale | Caucasian | 63 | 6 | 31.8 ± 9.2 | 30.6 ± 8.0 |  | 24.9 ± 7.4 | 31.7 ± 8.0 | 1.53 | 1.64 |
| Sayin | Caucasian | 35 | 3 | 22.74 ± 0.78 | 21.73 ± 0.56 |  | 22.80 ± 1.23 | 22.76 ± 1.33 | 1.59 | 1.56 |
| Tarkun | Caucasian | 57 | 6 | 23.46 ± 3.06 | 22.9 ± 2.97 |  | 23.71 ± 3.9 | 24.1 ± 3.7 | 1.53 | 1.54 |
| Vgontzas | Caucasian | 59 | 5 | 38.7 ± 1.4 | 36.9 ± 1.0 |  | 29.6 ± 0.9 | 35.7 ± 1.0 | - | - |
| Moran | Australia | 37 | 5 | 35.7 ± 5.8 | 35.5 ± 5.1 |  | 31.7 ± 6.2 | 37.1 ± 4.7 | 1.89 | 1.65 |
| Olszanecka | Caucasian | 73 | 6 | 35.6 ± 5.7 | 36.1 ± 5.5 |  | 27.4 ± 6.5 | 31.0 ± 5.9 | 0.9 | 1.71 |
| Jakubowska | Caucasian | 58 | 6 | 35.6 ± 5.7 | 36.1 ± 5.5 |  | 27.4 ± 6.5 | 31.0 ± 5.9 | 1.65 | 2.87 |
| Arikan | Caucasian | 69 | 6 | 21.48 ± 6.50 | 20.90 ± 6.04 |  | 22.82 ± 5.53 | 24.64 ± 4.22 | 1.48 | 1.98 |
| Samy | Asia | 91 | 6 | 22.7 ± 0.6 | 22.5 ± 0.5 |  | 28.7 ± 5.2 | 26.9 ±5.4 | 2.29 | 1.81 |
| Samy | Asia | 92 | 6 | 32.2 ±1.1 | 31.4 ± 1.2 |  | 28.3 ±4.2 | 27.5 ± 5.1 | 3.04 | 2.13 |
| Soares | Caucasian | 90 | 6 | 22.7 ± 3.3 | 23.1 ± 3.2 |  | 24.5 ± 3.8 | 24.5 ± 5.1 | 1.04 | 1.54 |
| Ilie | Caucasian | 77 | 5 | 28.41 ± 5.97 | 25.87 ± 6.89 |  | 23.11 ± 4.14 | 23.06 ± 5.37 | 1.01 | 1.03 |
| Victor | Caucasian | 82 | 6 | 22.2 ± 2.6 | 21.8 ± 2.6 |  | 24.2 ± 7.3 | 26.0 ± 5.0 | 1.73 | 1.8 |
| Xiong | Asia | 136 | 3 | 22.988 ± 6.566 | 20.816 ± 2.353 |  | 29.526 ± 2.818 | 30.479 ± 3.957 | - | 1.765 |
| Choi | Asia | 70 | 7 | 20.71 ± 2.35 | 19.88 ± 1.56 |  | 23.27 ± 4.33 | 24.58 ± 2.72 | 1.52 | 3.12 |
| Wang | Asia | 70 | 6 | 21.6 ± 2.6 | 21.7 ± 1.9 |  | 25.7 ± 4.5 | 26.8 ± 4.7 | 1.39 | 2.79 |
| Lee | Asia | 40 | 5 | 21.9 ± 2.0 | 21.9 ± 2.0 |  | 24 ± 5 | 24 ± 4 | 1.73 | 2.27 |
| Lee | Asia | 40 | 5 | 27.4 ± 1.9 | 27.3 ±1.7 |  | 26 ± 7 | 24 ± 5 | 1.21 | 1.95 |
| Li | Asia | 34 | 6 | 26.72 ± 1.37 | 26.71 ± 0.70 |  | 24.63 ± 1.19 | 25.44 ± 0.53 | 1.51 | 1.82 |
| Pawelczak | Asia | 35 | 3 | >25 | >25 |  | 15.2 ± 1.84 | 14.08 ± 1.7 | 1.05 | 1.95 |
| Thathapudi | Asia | 408 | 6 | 27.12 ± 4.93 | 23.4 ± 3.2 |  | 28 ± 3.6 | 28 ± 5.1 | 2.59 | 4.39 |
| Agacayak | Caucasian | 30 | 5 | 20.3 ± 2.2 | 20.4 ± 2 |  | 24.4 ± 4 | 26 ± 3 | - | 1.27 |
| Agacayak | Caucasian | 30 | 5 | 27.3 ± 2 | 27 ± 1 |  | 28 ± 4 | 30 ± 4.5 | - | 2 |
| Souza | Caucasian | 18 | 4 | <25 | <25 |  | - | - | 2.49 | - |
| Souza | Caucasian | 22 | 4 | >25 | >25 |  | - | - | 2.63 | - |
